# 6-Formylindolo (3,2-b)carbazole (FICZ) enhances retinoic acid (RA)-induced differentiation of HL-60 myeloblastic leukemia cells

**DOI:** 10.1186/1476-4598-12-39

**Published:** 2013-05-09

**Authors:** Rodica P Bunaciu, Andrew Yen

**Affiliations:** 1 Department of Biomedical Sciences, Cornell University, Ithaca, NY 14853, USA

**Keywords:** Retinoic acid (RA), 6-Formylindolo(3,2-b)carbazole (FICZ), Differentiation, Neutrophil, HL-60

## Abstract

**Background:**

The aryl hydrocarbon receptor (AhR) ligand 6-Formylindolo(3,2-b)carbazole (FICZ) has received increasing attention since its identification as an endogenous AhR ligand and a photoproduct of tryptophan. FICZ and its metabolites have been detected in human fluids. We recently reported that AhR promotes retinoic acid (RA)-induced granulocytic differentiation of HL-60 myeloblastic leukemia cells by restricting the nuclear abundance of the stem cell associated transcription factor Oct4. The standard clinical management of acute promyelocytic leukemia (APL) is differentiation induction therapy using RA. But RA is not effective for other myeloid leukemias, making the mechanism of RA-induced differentiation observed in a non-APL myeloid leukemia of interest. To our knowledge, this is the first study regarding the influence of FICZ on RA-induced differentiation in any type of leukemic blasts.

**Methods:**

Using flow cytometry and Western blotting assays, we determined the effects of FICZ on RA-induced differentiation of HL-60 human leukemia cells. All experiments were performed in triplicate. The groups RA and FICZ + RA were compared using the Paired-Samples *T*-Test. Western blot figures present the typical blots.

**Results:**

We demonstrate that FICZ enhances RA-induced differentiation, assessed by the expression of the membrane differentiation marker CD11b; cell cycle arrest; and the functional differentiation marker, inducible-oxidative metabolism. FICZ causes changes in signaling events that are known to drive differentiation, and notably augments the RA-induced sustained activation of the RAF/MEK/ERK axis of the mitogen-activated protein kinase (MAPK) cascade. FICZ also augments expression of the known MAPK signaling regulatory molecules c-Cbl, VAV1, pY458 p85 PI3K, Src-family kinases (SFKs), and IRF-1, a transcription factor associated with this putative signalsome that promotes RA-induced differentiation. Moreover, FICZ in combination with RA also increases expression of AhR and even more so of both Cyp1A2 and p47phox, which are known to be transcriptionally regulated by AhR. pY1021 PDGFRβ, a marker associated with retinoic acid syndrome was also increased.

**Conclusions:**

Our data suggest that FICZ modulates intracellular signaling pathways and enhances RA-induced differentiation.

## Background

Retinoic acid (RA) induces leukemic cell differentiation in a process that depends on AhR [1]. AhR overexpression drives differentiation [1]. This motivates interest in the effects of an endogenous AhR ligand on this process. AhR is a ligand activated receptor. There are two intensely studied AhR functions, both being ligand dependent. AhR is a basic helix-loop-helix/Per-Arnt-Sim (bHLH-PAS) transcription factor [2], and also an adaptor in the cullin 4B ubiquitin ligase complex [3]. It has been found to be expressed in all tissues analyzed. It is present in the cytosol and in the nucleus. Its transcriptional activity is the most studied, especially its regulation of detoxification enzymes such as cytochrome P450 [4]. The RAR/RXR and AhR pathways are known to crosstalk. For example, they compete for the silencing mediator of retinoid and thyroid receptors (SMRT) protein [5]. Consistent with various molecular indications of crosstalk, the two pathways can give rise to similar pathologies. For example, teratogenic effects such as cleft palate and hydronephrosis can be induced by retinoids [6] and also by an AhR agonist, 2,3,7,8-tetrachlorodibenzo-p-dioxin (TCDD) [7]. They can also contribute to common developmental processes. For example, in fish, RA and its receptors are required both for AhR transcription and embryonic development of blood vessels and bones [8]. AhR can thus regulate RA effects, as well as vice versa, but the mechanisms are not well understood.

Recently, several papers reported that the AhR gene can act as a tumor suppressor in the absence of xenobiotics. AhR has been shown to have a role in propelling breast cancer [9] and liver cancer [10] cell differentiation. AhR knockout mice injected with the liver tumor initiator diethylnitrosamine (DEN) have increased liver tumor formation and growth, with increased cell proliferation, inflammatory cytokine expression and DNA damage compared to wild type mice treated with DEN or untreated mice [10]. Moreover, the AhR knockout mice have increased cecal carcinogenesis [11]. Certain AhR antagonists promote hematopoietic stem cell proliferation [12]. The full molecular mechanism of AhR-dependent tumor suppressing activity is far from being elucidated; however, some details are emerging. Historically, the most studied function of AhR is its transcriptional activity elicited by xenobiotics. Recently it has become apparent that xenobiotics and endogenous ligands have different transcriptional properties, leading to opposite outcomes. For example, it was proposed that transient AhR transcriptional activity, characteristic of endogenous ligands such as 6-Formylindolo(3,2-b)carbazole (FICZ), is essential for the role of AhR in stem/progenitor cell homeostasis, whereas prolonged transcriptional activation is induced by exogenous ligands, such as TCDD, a well known carcinogen [13]. The more recently emerging role of AhR in protein degradation via CUL4B/AhR-mediated ubiquitylation and consequently cancer suppression is also of potentially related significance [11]. While the mechanisms are not yet clear, it appears that depending on the model system and on the ligand used, AhR can drive transformation or differentiation.

We have previously shown that AhR propels RA-induced differentiation of human myeloblastic leukemia cells by downregulating the nuclear transcription factor, Oct4 [1]. Oct4 is a Yamanaka-Thomson factor controlling stem cells [14-16]. This process depends on MAPK signaling. This motivates interest in the effect of endogenous AhR ligands, such as FICZ, on the MAPK pathway and its associated signaling events known to drive RA-induced differentiation. Unlike transcription, the effects of FICZ on signaling are less explored and remain to be better described.

One well studied model of leukemic cell differentiation is HL-60. HL-60 is a human myeloblastic leukemia cell line that is lineage uncommitted and capable of granulocytic or monocytic differentiation in response to different agents. HL-60 is a NCI-60 line, a set of standard cell lines, used for example in drug testing. It has been extensively used as a model for pharmacologically induced differentiation. HL-60 cells undergo granulocytic differentiation with G0/G1 growth arrest when treated with RA. This process requires sustained activation of MAPK signaling along the RAF/MEK/ERK axis [17], and a cascade of signaling regulatory events involving Src-family kinases, c-Cbl, VAV1, PI3K, and IRF-1 [18-22]. During RA-induced differentiation, ectopic expression of interferon regulatory factor 1 (IRF-1) [21] and c-Cbl [19] have been shown to enhance ERK 1/2 activation and promote RA-induced differentiation and G0/G1-arrest. The VAV1 guanine nucleotide exchange factor implicated in myelopoiesis also was reported to promote RA-induced granulocytic differentiation [23].

The present study demonstrates that FICZ is able to augment RA-induced differentiation. FICZ increases the amount and activation of key components of the MAPK signaling cascade known to drive differentiation, and this signaling modulation is consistent with a ligand bound AhR dependence as demonstrated by using the classical pharmacological AhR agonist β-naphthoflavone (β-NF) and antagonist α-naphthoflavone (α-NF). These had positive and negative effects on the signaling events consistent with their AhR agonist vs. antagonist activity. The findings suggest a novel potential mechanism of collaboration between RA and FICZ during RA-induced differentiation of t(15;17) negative leukemic blasts.

## Results and discussion

The capability to prevent and treat leukemia depends upon understanding the molecular underlying mechanisms of (a) pathogenesis, (b) induction of differentiation and apoptosis and (c) resistance to therapy. Multiple pathways are involved in each of these three aspects; however the aryl hydrocarbon receptor (AhR) is strikingly involved in all three of the above mentioned phenomena. We have shown that during RA-induced differentiation, AhR propels differentiation [1]. We now sought evidence on whether FICZ, an endogenous AhR ligand in humans, affects RA-induced leukemic cell differentiation.

### FICZ augments RA-induced differentiation markers

To determine if FICZ influenced RA-induced differentiation, HL-60 cells were treated with both agents either alone or in combination, and consequential occurrence of differentiation markers was measured. RA-induced granulocytic differentiation is characterized by the appearance of several phenotypic differentiation markers. These include: cell surface CD11b, cell cycle arrest in G0/G1, and inducible respiratory burst - a classical functional differentiation marker that is a characteristic response of mature myeloid cells to bacterial cell components. FICZ (100 nM) by itself had no effect on these markers. Co-administered with RA, FICZ enhanced the induced expression of these markers compared to RA alone.

**Figure 1:**
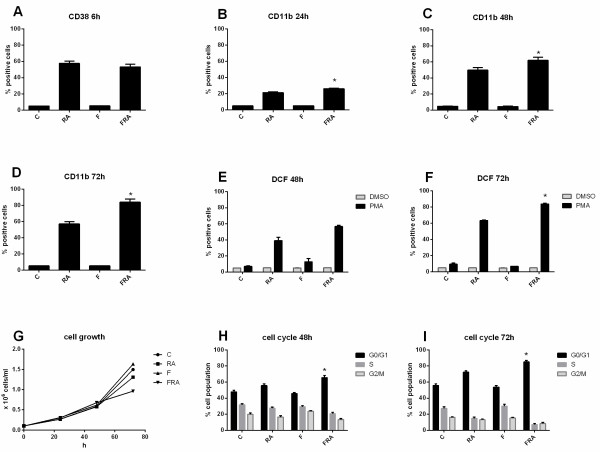
**FICZ augments RA-induced differentiation.** HL-60 cells were untreated (C, control) or treated with FICZ, RA, or RA plus FICZ for indicated times. FICZ augments RA-induced expression of differentiation markers. **A**. CD38 expression (assessed by flow cytometry with APC-conjugated antibody) at 6 h post treatment is not significantly modulated either by FICZ alone or in combination with RA. **B**. CD11b expression (assessed by flow cytometry with APC-conjugated antibody) was increased by the combination therapy compared to RA alone after 24 h (p = 0.012), **C**. 48 h (p = 0.042) and **D**. 72 h (p = 0.0029) of treatment. Flow cytometric assay of live cells was carried out setting the logical gate to exclude 95% of the untreated cells during CD38 and CD11b detection. **E**. Superoxide metabolites from inducible oxidative metabolism were measured using flow cytometry of DCF stained cells. Respiratory burst is not significantly enhanced at 48 h by combination therapy compared to RA alone (p = 0.08). **F**. Respiratory burst, measured by flow cytometry of DCF stained cells, is significantly enhanced at 72 h by co-treatment compared to RA alone (p = 0.001). **G**. Cell density was measured for control and treated cells at progressive times, 0, 24, 48 and 72 h. Cell growth shows that FICZ at the concentration used has no toxic effect. FICZ by itself does not decrease the cell number, however it enhances RA-induced growth inhibition. **H**. Cell cycle distribution was measured by flow cytometry of propidium iodide stained nuclei. The percent of cells in G0/G1 is shown. G0/G1 cell-cycle arrest was propelled by co-treatment compared with RA alone both at 48 h (p = 0.009) and **I**. 72 h(p = 0.035) as shown by flow cytometry of DNA-stained nuclei.

Cells were untreated or treated with 1 μM RA with or without 100 nM FICZ. Expression of the CD38 and CD11b cell surface differentiation markers, the respiratory burst (measured as inducible reactive oxygen species) and the percentage of cells with G0/G1 DNA were measured by flow cytometry (Figure 1). CD38 is an early cell surface differentiation marker. At 6 h, FICZ alone did not induce CD38 expression. Likewise, FICZ did not affect RA-induced CD38 expression at this early time (Figure 1A). CD11b is the alpha subunit of the integrin receptor and is a differentiation marker that typically appears with slower kinetics than CD38 in RA-treated cells. For CD11b expression, the percentage of cells that were positive was higher for cells treated with RA plus FICZ compared to RA alone, namely 26% versus 21%, p = 0.012 after 24 h (Figure 1B), 62% versus 50%, p = 0.042, after 48 h (Figure 1C) and 84% versus 57%, p = 0.0029, after 72 h (Figure 1D). The flow cytometry raw data and mean fluorescence index for a representative experiment (48 h and 72 h) are presented in Additional file 1: Figure S1. Cells treated with FICZ alone showed no CD11b expression – like untreated controls.

Inducible oxidative metabolism is a functional marker of further differentiation that is characteristic of mature cells. This mature functional differentiation marker was also enhanced in cells treated with FICZ plus RA compared to RA alone. At 48 h, FICZ plus RA treated cells were 57% positive compared to 39% for cells treated with RA alone with a p = 0.08 (Figure 1E), and by 72 h 84% of FICZ plus RA treated cells were positive versus 63% of RA treated cells with a p = 0.001 (Figure 1F).

G0/G1 cell cycle arrest is a characteristic of differentiation. RA caused an increase in the relative number of G0/G1 cells and an associated reduction in S phase cells. Addition of FICZ with RA enhanced this effect (Figure 1H and 1I), consistent with the enhanced phenotypic shift. At 48h (Figure 1H), 48% cells were in G0/G1 phase for untreated cells, and 56% for RA treated cells, p < 0.0001. At 72 h (Figure 1I), the proportions were 56% and 72% for untreated and RA treated respectively (p < 0.001). FICZ alone had a slightly lower proportion of cells in G0/G1 compared to untreated cells (46% vs. 48%, p = 0.2 at 48 h, and 54% vs. 56%, p = 0.01 at 72 h). For cells treated with FICZ plus RA compared to RA alone, the percentage of cells with G0/G1 DNA was 66% compared to 56%, p < 0.0001, after 48 h; and 85% versus 72%, p < 0.0001, after 72 h. Growth curves (Figure 1G) were consistent with the cell cycle phase distribution changes. FICZ alone did not significantly affect, although slightly increased, the cell density compared with control (1.5 ± 0.08 × 10^6^ cells/ml for control, and 1.6 ± 0.1 × 10^6^ cells/ml for FICZ at 72h post-treatment). FICZ in combination with RA lowered the cell densities compared to RA alone (0.96 ±0.15 × 10^6^ cells/ml for the combination treatment and 1.3 ± 0.1 × 10^6^ cells/ml for RA alone at 72 h post-treatment) consistent with the G0/G1 data. FICZ thus enhances RA-induced CD11b expression, inducible oxidative metabolism, and G0/G1 arrest, but does not modulate these parameters by itself in the absence of RA. FICZ caused no evident toxicity, evaluated by trypan blue exclusion or population growth, and FICZ-treated cells had similar cell cycle phase distribution and growth curves as untreated control cells.

Given the positive effects of FICZ on RA-induced differentiation, we sought evidence that the FICZ as presented in this context could regulate the transcriptional activity of AhR by determining its effects on two classical AhR transcriptionally regulated targets: Cyp1A2 and p47phox.

### FICZ augments the expression of classical AhR transcriptionally regulated genes

**Figure 2:**
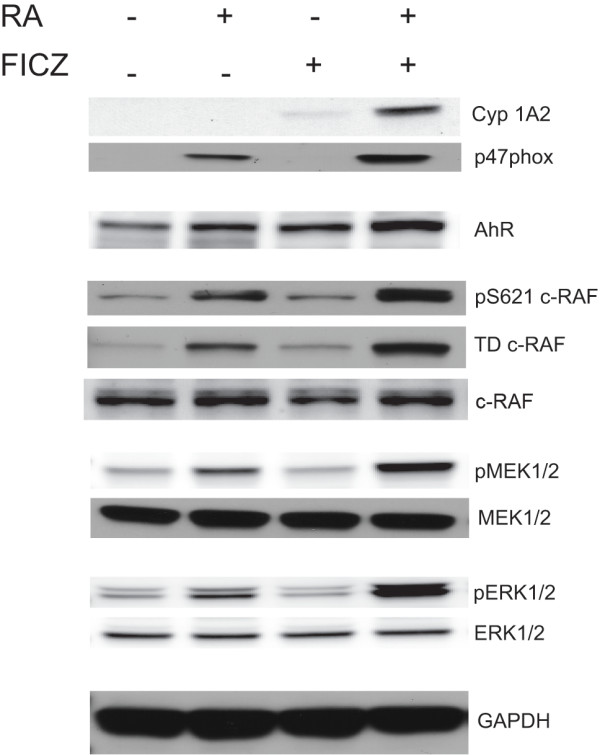
**FICZ upregulates: AhR, AhR target genes and MAPK cascade components in the presence of RA.** Cell lysates collected 48 h after initiation of the experiments were resolved on 12% polyacrylamide gels. 25 μg protein was loaded per well. In RA-treated cells, FICZ upregulates the amount of Cyp1A2, and p47phox, AhR protein expression and pERK1/2, pMEK1/2 and pS621 RAF phospho-protein in the presence of RA compared to cells treated with RA alone. Like pS621 RAF, terminal domain phosphorylated RAF is also upregulated. Total ERK1/2, MEK1/2, RAF are unchanged. The loading control was GAPDH.

The expression of cytochrome P450 1A2 (Cyp1A2), neutrophil cytosolic factor-1 (p47phox), and aryl hydrocarbon receptor (AhR), were analysed after 48 h of treatment with FICZ, RA or their combination using Western blotting (Figure 2). We found that relative levels of Cyp1A2 and p47phox proteins were clearly increased by the combination therapy (lane 4) compared with untreated control cells (lane 1). Addition of FICZ to RA (lane 4) also increased Cyp1A2 and p47phox expression compared to RA only-treated cells (lane 2). Cyp1A2, an endogenous reporter of classical AhR driven transcriptional activation thus behaved as expected. RA alone did not induce Cyp1A2 expression, and FICZ induced it both alone and more strongly with RA. The protein p47phox, a NADPH oxidase subunit of the complex producing the respiratory burst, was also reported to be under AhR transcriptional control [24]. In contrast to Cyp1A2, the changes in p47phox expression depended on the presence of RA. FICZ was able to upregulate p47phox expression only in RA-treated cells. This was anticipated since p47phox expression is a characteristic of mature myeloid cells, and RA is needed to cause granulocytic differentiation. AhR expression was modestly increased by RA plus FICZ (lane 4) compared to RA alone (lane2). Previous reports showed that AhR protein expression is augmented by treatment with RA [1] or FICZ alone [25] and we confirmed this (lane 2 vs lane 1 or lane 3 vs lane 1). FICZ thus increases the expression of genes that are classical targets of AhR. While the present results are consistent with action through AhR, there could be a variety of other transcription factors that also contribute to the FICZ induced effects observed.

It is now well established that a transient activation of the MAPK signaling cascade elicits cell proliferation, whereas prolonged activation leads to differentiation [17,26]. In particular RAF activation is known to drive RA-induced differentiation [27]. We therefore assessed the effects of FICZ on the MAPK cascade, specifically the RAF/MEK/ERK axis that is activated during RA-induced differentiation.

### FICZ augments RA-induced MAPK signaling cascade

MAPK signaling during RA-induced differentiation utilizes c-RAF activation, specifically pS621 c-RAF phosphorylation, which is necessary to induce terminal granulocytic differentiation [27]. Western blot analysis confirms that FICZ and RA co-treatment enhances c-RAF activation (Figure 2) compared to RA alone. FICZ alone had no effect. The same behavior is true for the other two components of the MAPK cascade: pMEK1/2 and pERK1/2. Total amounts of c-RAF, MEK, and ERK in contrast were not upregulated in this time frame (48 h) by FICZ or FICZ plus RA. The data thus indicate FICZ regulates intracellular signaling events, but not c-RAF, MEK or ERK abundance – such as might occur through AhR regulated transcription or protein stability. Interestingly, FICZ and RA co-treatment also resulted in increased phospho-c-RAF pS289/296/301 (TD RAF) compared to RA alone. This C-terminal domain of c-RAF is phosphorylated during RA-induced differentiation and is thought to be part of a putative feedback loop characterizing hyperactive MAPK signaling needed for differentiation [18]. In other contexts, it is also known to be phosphorylated by ERK1/2 [28] and can make the c-RAF molecule unresponsive to further stimulation [29], suggesting that this phosphorylation event may have a diversity of potential effects dependent on context. FICZ thus augments the RA-induced activation of the RAF/MEK/ERK axis. The enhanced activation is consistent with the occurrence of enhanced differentiation attributed to FICZ above.

The MAPK signalsome that drives RA-induced differentiation is known to contain a number of regulatory molecules that propel differentiation. We thus sought evidence of their involvement consequential to FICZ. Interestingly, the signalsome has been found to contain the transcription factor IRF-1 which has also been found to propel RA-induced differentiation [21].

### MAPK signaling cascade modulation by FICZ is consistent with modulation of other signalsome regulatory molecules of the RA-induced differentiation process

**Figure 3:**
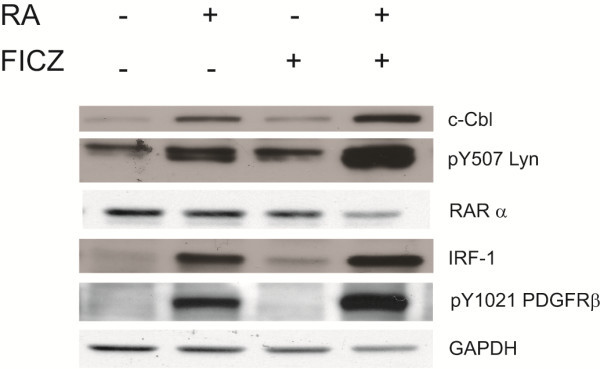
**FICZ upregulates RA-dependent signaling intermediates.** Cell lysates collected at 48 h time point were resolved on 12% polyacrylamide gels. 25 μg protein was loaded per well. FICZ upregulates the amount of c-Cbl and IRF-1 expression and pY507 Lyn and pY1021 PDGFRβ phospho-protein. Total RARα expressed is not upregulated, nor is the GAPDH loading control.

c-Cbl and IRF-1 have been previously shown to be instrumental in RA-induced differentiation; specifically, increased expression propelled differentiation [21]. Cells were treated with RA or FICZ alone or in combination, and expression of c-Cbl, pY507 Lyn, RARα, IRF-1 and pY1021 PDGFRβ was measured. FICZ augments the RA-induced increases in c-Cbl and IRF-1 (Figure 3). This is consistent with previous results where we have shown that AhR expression induced IRF-1, and IRF-1 physically interacted with c-Cbl [21]. To confirm that the increases in amount of protein that we observe are not attributable to a general nonspecific increase in protein synthesis, we have confirmed that the amount of RARα or GAPDH did not increase. Lyn is a member of the Src-family kinases (SFKs), and its binding to c-RAF in RA treated cells is enhanced by the SFK inhibitor PP2, which enhanced RA-induced differentiation [18]. We reported that a scaffolding function of Lyn – not its kinase activity – was important for RA-induced differentiation [18]. Phosphorylation of Lyn at Y507 increases autoinhibition of its kinase activity [30]. RA increases the amount of pY507 Lyn and addition of FICZ augments this, again consistent with a role of FICZ in enhancing RA-induced effects on signaling molecules. We also assessed pY1021 PDGFRβ expression. pY1021 PDGFRβ is potentially significant as a marker of neutrophil hyperactivation, consistent with the report that pY1021 PDGFRβ is a marker of retinoic acid syndrome [31]. It was also up regulated by RA, and addition of FICZ to the RA further enhanced it. FICZ thus enhanced RA effects on a number of RA-targeted signaling regulatory molecules associated with induced differentiation.

We sought evidence to corroborate the putative action of FICZ through AhR to drive signaling effects by using other known AhR agonists and antagonists.

### The effects of other AhR ligands on signaling

**Figure 4:**
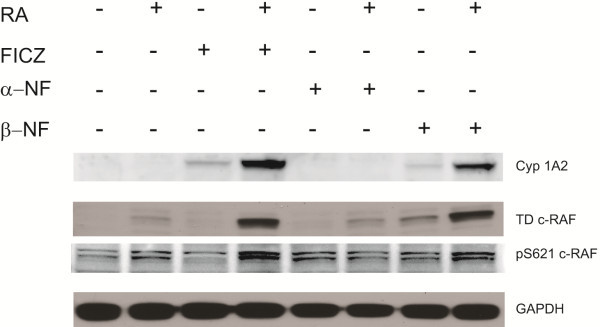
**AhR ligands modulate phosphorylation of RAF in the MAPK signaling cascade.** Cell lysates collected 48 h after initiation of the experiments were resolved on 12% polyacrylamide gels. 25 μg protein was loaded per well. β-NF upregulates the amount of Cyp1A2, TD RAF and pS621RAF proteins in the presence of RA in a similar fashion to FICZ (comparing similarity of lanes 4 and 8), whereas α-NF does not. GAPDH is the loading control.

The ability of FICZ to modulate signaling molecules in the context of RA-treated cells is novel. FICZ is an endogenous AhR ligand. This motivated interest in determining if other AhR ligands also had consistent effects on signaling. Two well characterized exogenous AhR ligands were used: an AhR antagonist, α-NF, and an agonist, β-NF, at a concentration of 1 μM each. Cells were treated with RA, FICZ, α-NF or β-NF as shown in the figures. The effects on Cyp1A2, TD RAF and pS621 c-RAF were measured by Western blotting as shown in Figure 4. Cyp1A2 is a classical responder to AhR activation and was used to confirm the ability of the ligands to activate AhR or not. FICZ increases Cyp1A2 expression and behaves as an AhR agonist as expected. At the concentration used β-NF elicits Cyp1A2 expression also, whereas α-NF does not, consistent with their known roles as an AhR agonist or antagonist, respectively. RA augments the effects of the AhR agonists (FICZ or β-NF), but not the antagonist. This suggests cooperativity between RA and the agonists (with lanes 4 and 8 of Figure 4 having the strongest signals). We next determined if there were corresponding cooperative effects on signaling events believed to drive RA-induced differentiation. RA-induced upregulation of the C-terminal domain phosphorylated RAF (TD-RAF), and this is enhanced by the AhR agonists (FICZ and β-NF), but not by the antagonist (α-NF). There are similar but more subtle effects on the expression of pS621 c-RAF. RA and the agonists again cooperate, and pS621 c-RAF expression is greater for RA plus agonist than RA alone. Both the C-terminal domain and S621 c-RAF phosphorylations are characteristic of RA-induced signaling. Hence the TD RAF and pS621 c-RAF responses to RA are augmented by AhR agonists.

**Figure 5:**
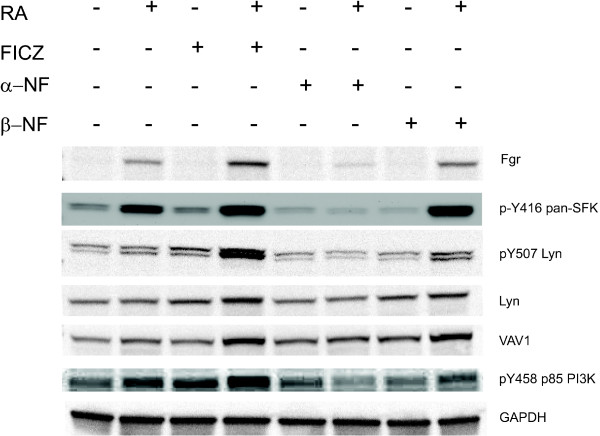
**FICZ and other AhR ligands modulate MAPK signaling cascade regulatory molecules.** Cell lysates collected after 48 h of RA treatment were resolved on 12% polyacrylamide gels. 25 μg protein was loaded per well. Similarly to FICZ, β-NF upregulates the amount of Fgr, Lyn, VAV1 protein and p-Y416 pan-SFK, pY507 Lyn, and pY458 p85PI3K phospho-protein, whereas the antagonist does not upregulate those markers Comparing lanes 4 and 8 FICZ and β-NF, the two AhR agonists, have apparently similar signaling profiles in RA-treated cells, which is not shared by α-NF, an AhR antagonist, lane 6). The agonists by themselves without RA do not have this effect. GAPDH is the loading control.

The RA-regulated RAF/MEK/ERK axis has been found to be associated with a number of signaling regulatory molecules in a putative signalsome that propels RA-induced differentiation. Prominent MAPK signaling regulators in the RA-induced signaling cascade leading to RA-induced differentiation that have emerged are: Src-family kinases [18], VAV1 [23,32] and PI3K [22]. Cells were treated with RA or the antagonist or agonists singly or in combination with RA as above and the expression of these targeted signaling molecules was measured. The protein levels and activation of these signaling molecules are modulated during RA-induced differentiation by AhR ligands (Figure 5). Fgr, a SFK, is one of the most responsive of these proteins. RA-induced upregulation of Fgr is enhanced by FICZ and β-NF, AhR agonists, but is crippled by α-NF, an AhR antagonist. The AhR ligands by themselves had no discernible effect on expression, indicating an AhR role dependent on the RA-induced context. Consistent with this, the enzymatically active form of SFKs discerned by probing with a p-Y416 pan-Src-family kinase antibody also responded similarly to the AhR ligands as seen for Fgr expression. The results are consistent with earlier observations for these cells that the SFKs are progressively activated by tyrosine phosphorylation after RA treatment and reach maximum phosphorylation 48 hours post treatment [33]. Taken together, these observations are thus consistent with a role for these kinases in driving differentiation. Interestingly, the pY507 Lyn is also regulated likewise by RA and the AhR agonists and antagonist (lanes 4 and 8 Figure 5 show the strongest signal again). This phosphorylation site has been implicated with a negative role in another context [34]. This is consistent with a previous suggestion that Lyn performs a scaffold function important for the signalsome to drive differentiation [18]. The total amount of Lyn, the VAV1 guanine nucleotide exchange factor and pY458 p85 PI3K also exhibit a similar enhancement after RA plus FICZ combination treatment compared to RA alone. However the effects of α-NF and β-NF on total Lyn, VAV1, and pY458 p85 PI3K are minor compared to the modulation of Fgr, p-Y416 pan-SFK and pY507 Lyn. In sum, our results show that the effects of the β-NF agonist and α-NF antagonist on certain signaling molecules that are implicated as part of the RA-regulated MAPK signaling complex are consistent with a role for AhR in driving FICZ augmentation of RA-induced differentiation via effects on components of the putative signalsome. However, some components are more responsive than others.

## Conclusions

Taken together our results show that FICZ enhances RA-induced differentiation as evidenced by CD11b membrane receptor expression, inducible respiratory burst, G0/G1 cell cycle arrest and growth curves. Upregulation of signaling molecules previously shown to drive RA-induced differentiation was enhanced by addition of FICZ. Specifically, FICZ augments RAF/MEK/ERK axis MAPK signaling, Src-family kinase activation and the RA-dependent upregulation of VAV1, c-Cbl, pY458 p85PI3K, and IRF-1 expression. Interestingly, in the case of PDGFR which differentially regulates different aspects of RA-induced differentiation [35], PDGFRβ phosphorylation at Tyr 1021, a response associated with mimicking retinoic acid syndrome in this model [31], was also enhanced by the RA plus FICZ combination treatment compared to RA treatment alone.

**Figure 6:**
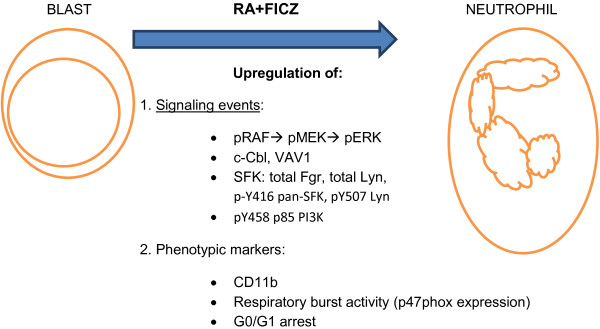
Summary figure.

AhR is involved in both promoting and inhibiting proliferation. AhR has been implicated with historically well-known pro-proliferative functions. For example, benzene, an AhR agonist, is known to induce both leukemia and multiple myeloma [36]. But AhR has also been shown to have a role in propelling breast cancer [9] and liver cancer [10] cell differentiation. The present results suggest that RA may set a context for AhR to act in an anti-proliferation pro-differentiation capacity. Here we provide evidence for a novel way of using an endogenous AhR ligand to enhance RA-induced differentiation associated with the unanticipated modulation of components of the MAPK and Src-family kinase signaling machine/signalsome thought to drive RA-induced differentiation (summarized in Figure 6).

The present results suggest cooperative crosstalk between the RA and FICZ elicited pathways in driving differentiation. How this occurs molecularly is a matter of conjecture that will require further experimental elucidation. There are numerous pathways that RA and FICZ are able to elicit. The most studied are RAR/RXR and AhR transcriptional regulation pathways. There are several ways those pathways are known to crosstalk. (1) For example, they compete for transcriptional co-activators/repressors, such as SMRT protein [5,37]. However, in our case, the amount of SMRT that co-immunoprecipitates with AhR does not vary with different treatments (data not shown), suggesting that this is not the mechanism involved in this case. (2) RAR and AhR can also use the same coactivators, specifically SRC-1, steroid coactivator-1 [38,39]. (3) Retinoids are reported to be AhR ligands that can drive AhR/ARNT to xenobiotic response elements (XRE) and consequently regulate transcription [5]. (4) RAR and AhR pathways can also crosstalk by regulating the same transcription factor, notably the pro-proliferation transcription factor AP-1. RAR can physically bind either c-jun or c-fos resulting in a mutual inhibition of DNA-binding activity for both RAR and AP-1 [40]. AhR is also reported to inhibit AP-1 DNA binding activity [41]. (5) RAR and AhR regulation of transcription can depend on common transcription factors such as the COUP orphan receptors which are regulators of both AhR [42] and of RAR directed transcriptional activity [43]. There are thus a variety of ways that RA and AhR governed pathways can converge at the level of transcription.

While crosstalk at the level of transcriptional regulation is arguably the most prominently studied, non-nuclear/cytoplasmic interactions at the level of signaling are also indicated. RA itself can regulate MAPK related signaling molecules such as PKC [44,45] or c-RAF [46] as a lipid interacting molecule with a hydrophobic pocket [47,48]. AhR can also regulate pathways incorporating MAPK signaling molecules [1,49]. AhR has been found complexed with Src, a well-known MAPK signaling regulator [50]. And MAPK signaling has been shown to be a downstream effector for both RA and AhR, consistent with the possibility that RA and AhR integrate their cytoplasmic signaling through the MAPK axis [1,17]. AhR is also known to have a ubiquitin E3 ligase activity that can affect expression levels of other molecules, notably ER which we have reported can act as a membrane receptor – in addition to its historical nuclear function as a ligand activated transcription factor - that originates MAPK signaling relevant to RA-induced differentiation [51]. There are thus a number of possibilities for the mechanism of non-nuclear as well as nuclear crosstalk already suggested in the literature. The present results motivate interest in deciphering their roles in RA-induced differentiation augmented by FICZ.

RA has clinically been notably successful in inducing remissions, albeit transient, in APL, but has not been effective in other myeloid leukemias. APL is defined by the presence of the PML-RARα fusion protein resulting from the t(15;17) translocation that cytogenetically characterizes the disease, which is a FAB M3. There is thus potential interest from the therapeutic point of view of bringing RA differentiation induction therapy to non-APL FAB M2 or 1 disease. In particular mechanistic aspects of how a FAB M2 derived cell that is capable of RA-induced differentiation undergoes granulocytic differentiation and G0 cell cycle arrest may provide insights into how to drive differentiation in a non-APL cell. Such is HL-60, the currently used model derived from a myeloblastic leukemia. Hence means of driving RA-induced differentiation here may contribute insights of therapeutic relevance.

## Methods

### Cell culture and treatments

HL-60 human myeloblastic leukemia cells derived from the original patient isolate, a generous gift of Dr. Robert Gallagher, were grown in RPMI 1640 (Invitrogen, Carlsbad, CA) supplemented with 5% fetal bovine serum (Hyclone, Logan, UT) and 1x antibiotic/antimycotic (Sigma, St. Louis, MO) in a 5% CO_2_ humidified atmosphere at 37°C. The cells were cultured in constant exponential growth as previously described [52]. The experimental cultures were initiated at a density of 0.1 × 10^6^ cells/ml. Viability was monitored by 0.2% trypan blue (Invitrogen, Carlsbad, CA) exclusion and routinely exceeded 95%. All reagents were purchased from Sigma (St Louis, MO) unless otherwise stated.

For treatments, all-trans-retinoic acid (RA) (Sigma, St. Louis, MO) was added from a 5 mM stock solution in 100% ethanol to make a final concentration of 1 μM in culture. 6-Formylindolo(3,2-b)carbazole (FICZ) (Enzo Life Sciences, Exeter, United Kingdom), was added from a 100 μM DMSO stock to make a final concentration of 100 nM in culture. The concentration was chosen from an initial dose response experiment as the lower concentration yielding a phenotypic response when added with RA with no toxic effects. This corresponds to a frequently used concentration in the literature. α-naphthoflavone and β-naphthoflavone (both from Sigma, St. Louis, MO) were each used at a final concentration of 1 μM in culture. The stock solutions were 1 mM in DMSO. Similar to FICZ, there was no apparent toxicity of α-NF or β-NF at this dose discernible by proliferation rates, cell cycle distribution, or trypan blue exclusion.

### CD38, CD11b quantification

Expression of cell surface differentiation markers was quantified by flow cytometry. 1 × 10^6^ cells were collected from cultures and centrifuged at 1000 rpm for 5 min. Cell pellets were resuspended in 200 μl, 37°C, PBS containing 2.5 μl of allophycocyanin (APC) conjugated antibody for CD11b or CD38 (both from BD Biosciences, San Jose, CA). Following a 1 h incubation at 37°C cell surface expression levels were analyzed with a BD LSRII flow cytometer (BD Biosciences, San Jose, CA). APC is excited at 633 nm and emission collected with a 660/20 band pass filter. Undifferentiated control cells were used to determine the fluorescence intensity of cells negative for the respective surface antigen. The gate to determine percent increase of expression was set to exclude 95% of the control population.

### Respiratory burst quantification

Respiratory burst (a functional differentiation marker for mature myelo-monocytic cells) was measured by flow cytometry. 1 × 10^6^ cells were collected and centrifuged at 1000 rpm for 5 min. Cell pellets were resuspended in 500 μl 37°C PBS containing 5 μM 5-(and-6)-chloromethyl-2′,7′-dichlorodihydro–fluorescein diacetate acetyl ester (H2-DCF, Molecular Probes, Eugene, OR) and 0.2 μg/ml 12-o-tetradecanoylphorbol-13-acetate (TPA, Sigma, St. Louis, MO). H2-DCF and TPA stock solutions were made in DMSO (Sigma, St. Louis, MO) at concentrations of 0.2 mg/ml and 5 mM, respectively. A control group incubated in H2-DCF and DMSO only was included. Cells were incubated for 20 min at 37°C prior to analysis by flow cytometry. Oxidized DCF was excited by a 488 nm laser and emission collected with a 530/30 nm band pass filter. The shift in fluorescence intensity in response to TPA was used to determine the percent cells with the capability to generate inducible oxidative metabolites [36]. Gates to determine percent positive cells were set to exclude 95% of control cells not stimulated with TPA.

### Cell cycle quantification

1 × 10^6^ cells were collected by centrifugation and resuspended in 200 μl of cold propidium iodide (PI) hypotonic staining solution containing 50 μg/ml propidium iodine, 1 μl/ml Triton X-100, and 1 mg/ml sodium citrate (all from Sigma, St. Louis, MO). Cells were incubated at room temperature for 1h and their nuclei analyzed by flow cytometry (BD LSRII) using 488-nm excitation and emission collected with a 575/26 band-pass filter. Doublets were identified by a PI signal width versus area plot and excluded from the analysis [36].

### Protein detection by Western blot

2 × 10^7^ cells were lysed using 200 μL lysis buffer (Pierce, Rockford, IL) and lysates were cleared by centrifugation at 13,000 rpm for 30 min at 4°C. Equal amounts of protein lysates (25 μg) were resolved by SDS-PAGE gel electrophoresis, transferred to PVDF membranes and probed with antibodies. AhR (H211), c-Cbl (C-15) and p Y1021 PDGFRβ antibodies were from Santa Cruz Biotechnology (Santa Cruz, CA). IRF-1 and CD38 antibodies were from BD Biosciences (San Jose, CA). Antibodies to detect phospho-p44/42 MAPK (ERK1/2) (Thr202/Tyr204) (D13.14.4E), p44/42 MAPK (ERK1/2) (137F5), pS221 MEK1/2, MEK1/2, p-Y416 pan-SFK, Lyn, pY507 Lyn, p-PI3K p85(Y458)/p55 (Y199), Fgr, VAV1, p47phox, pS289/296/301 c-RAF (TD cRAF), RARα, and GAPDH, horseradish peroxidase anti-mouse and horseradish peroxidase anti-rabbit were from Cell Signaling (Danvers, MA, USA). pS621 c-RAF antibody was from Invitrogen (Carlsbad, CA). Cyp1A2 antibody was from Abcam (Cambridge, MA). ECL (GE Healthcare, Pittsburgh, PA) was used for detection.

### Statistical analysis

Statistical analyses were performed using GraphPad (GraphPad software, San Diego, CA). Means of treatment groups of interest were compared using the Paired-Samples *T* Test. The data represents the means of three repeats ± S.E.M. A p-value of < 0.05 was considered significant.

## Competing interests

The authors declare no conflict of interests.

## Authors’ contributions

RPB and AY participated in the design of the study. AY coordinated the studies. RPB carried out the experiments. RPB and AY wrote the final manuscript. All authors read and approved the final manuscript.

## Supplementary Material

Additional file 1: Figure S1 CD11b Mean Fluorescence Index (MFI) Shift. The flow cytometry raw data and mean fluorescence index for CD11b of a representative experiment (48 h and 72 h) are presented. CD11b expression was assessed by flow cytometry with APC-conjugated antibody. HL-60 cells were untreated (C, control) or treated with FICZ, RA, or RA plus FICZ. Cells treated with FICZ alone showed no CD11b expression – like untreated controls. Click here for file
