# Supplementary figures and images for: 6-Formylindolo (3,2-b)carbazole (FICZ) enhances retinoic acid (RA)-induced differentiation of HL-60 myeloblastic leukemia cells
Source: Mol Cancer. 2013 May 9;12:39. doi: 10.1186/1476-4598-12-39 (PMC3693992; doi:10.1186/1476-4598-12-39)

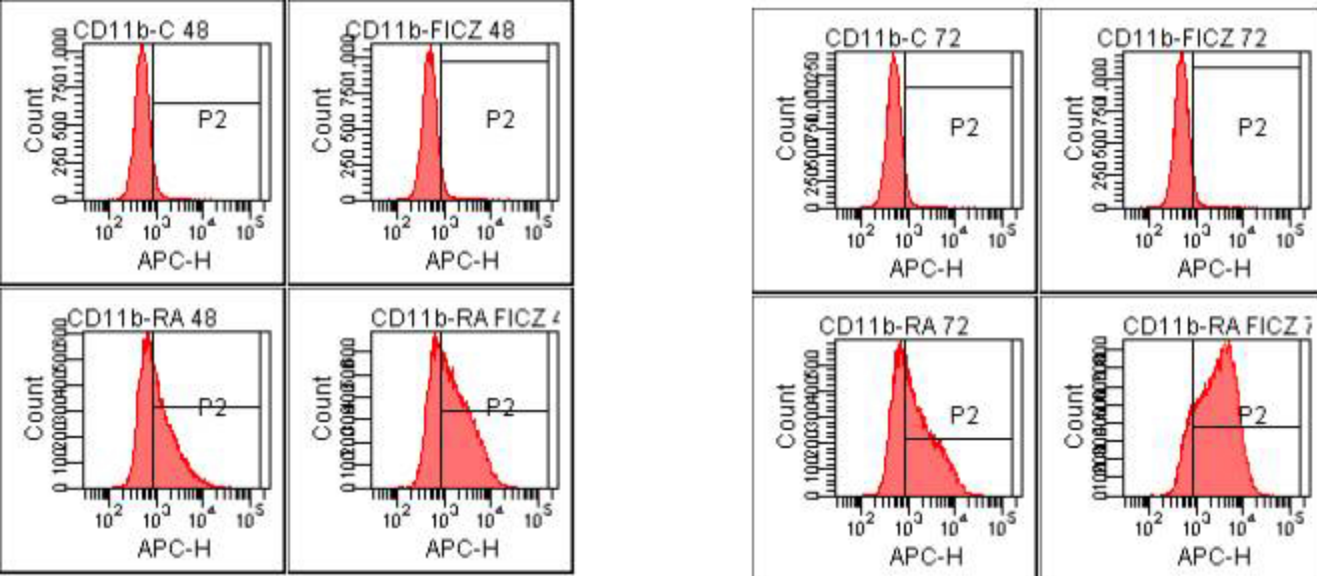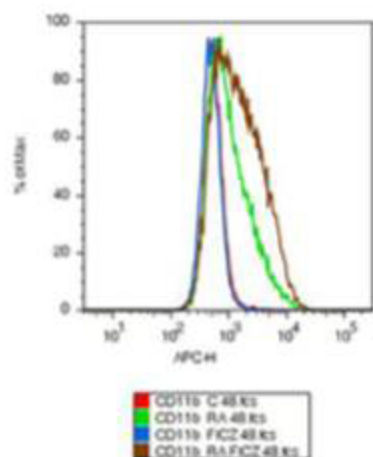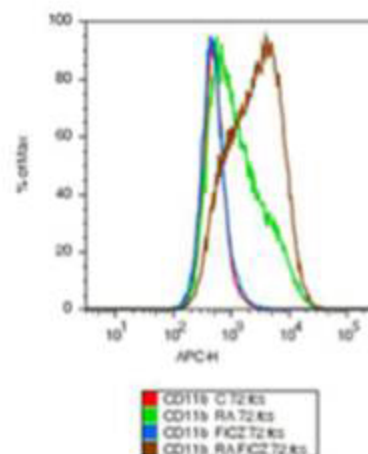

48h CD11b (Mean Fluorescence Index)

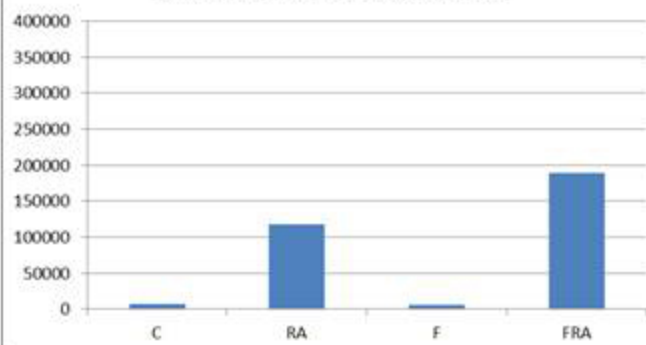

72h CD11b (Mean Fluorescence Index)

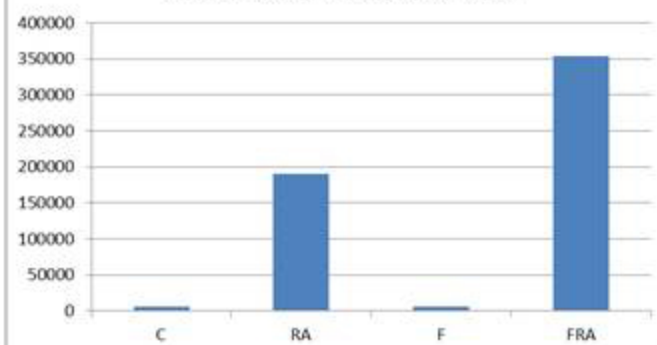

Supplement: Additional file 1: Figure S1 — CD11b Mean Fluorescence Index (MFI) Shift. The flow cytometry raw data and mean fluorescence index for CD11b of a representative experiment (48 h and 72 h) are presented. CD11b expression was assessed by flow cytometry with APC-conjugated antibody. HL-60 cells were untreated (C, control) or treated with FICZ, RA, or RA plus FICZ. Cells treated with FICZ alone showed no CD11b expression – like untreated controls. [file 1476-4598-12-39-S1.pdf]
